# A sensitive and rapid immunoassay for *Mycoplasma pneumoniae* in children with pneumonia based on single-walled carbon nanotubes

**DOI:** 10.1038/s41598-017-16652-3

**Published:** 2017-11-27

**Authors:** Ming Song, Ying Zhang, Shi Li, Chunsheng Zhang, Mingming Tao, Ying Tang, Zhuquan Jiang, Sulan Cai, Wei Xu, Weizhuo Xu

**Affiliations:** 1 Institute of Life Science and Bio-pharmaceutics, Shenyang Pharmaceutical University, Shenyang, 110016 China; 2 Shenyang Entry-Exit Inspection and Quarantine Bureau Technology Center, Shenyang, 110016 China; 3 Haihua College, Liaoning Normal University, Shenyang, 110167 China; 4 Benxi TestJet Biotechnology Co., Benxi, 117004 China; 5 The First Hospital of Jilin University, Changchun, 130021 China

## Abstract

*Mycoplasma pneumoniae*(MP) is a leading pathogen of respiratory infection, especially community-acquired pneumonia (CAP), in children worldwide. However, its diagnosis is frequently ineffective because bacterial culture and serology test are usually positive 1–2 weeks or more after the disease onset. To achieve a better detection efficiency, the single-walled carbon nanotubes(SWCNT) were coupled with the colloidal gold-monoclonal antibody immunochromatographic strips(CGIC). Interestingly, the SWCNT/CGIC assay allowed MP identification, with a detection limit of 1 × 10^2^ copies/ml. Using referenced throat swabs of 97 MP and 40 non-MP cases, the assay yielded 72.2% sensitivity, 100.0% specificity, 100.0% positive predictive value (PPV), 59.7% negative predictive value (NPV). In summary, our assay was far more effective than any conventional methods for the diagnosis of acute MP. The ease of use, rapid and stability further enhance its feasibility for clinical use on-site.

## Introduction

*Mycoplasma pneumoniae* (MP) is a respiratory bacterial pathogen causing upper and lower respiratory disease in humans of all ages. It is considered a major cause of pneumonia, especially in children of school age and in some cases can result in serious extrapulmonary sequelae^1,2^. MP was detected in 30% of pediatric community-acquired pneumonia (CAP), and in over 50% among children aged 5 years or older^3^. These infections are difficult to discern from other causes of pneumonia based on examination, symptoms, or chest X-ray findings, and treatment with routinely used β-lactam antibiotics is ineffective against such organisms that lack a cell wall, laboratory identification of the etiology is critical to establish the correct course of treatment^4,5^. There are no specific clinical, epidemiological, or laboratory findings that allow a definite diagnosis of Mycoplasma infection early in the clinical course. Thus, isolation of MP remains the gold standard as a diagnostic procedure for this infection^6^. Cultures of a throat swab or sputum specimens may show MP, but growth is rarely detected earlier than 1 week after the start of culture. Seroconversion or a 4-fold increase in the MP antibody titer on examination of acute and convalescent sera is also diagnostic. However, the confirmation of MP infection by such methods is generally too slow to be of practical use^7^. During the past years, many analytical techniques for quantitative determination of MP have been studied, including Enzyme-linked immunosorbent assays (ELISA) and Polymerase chain reactionanalysis (PCR), which are sensitive but typically require skilled operators, complex sample pretreatments, expensive instruments, and time-consuming, thus impairing their applications in detection of MP^8,9^.

Carbon nanomaterials(CNMs) have shown great potential in biomedical applications, mainly due to their unique chemical and physical properties^10,11^. Carbon nanotubes is one of the most widely used CNMs due to their physical and chemical stability as well as their high surface area-to-weight ratio^12,13^. In this study, we developed an ultrasensitive antigen assay based on the single-walled carbon nanotubes(SWCNT) coupled with the colloidal gold-monoclonal antibody immunochromatographic strips (CGIC). Then, a large sample size study was conducted to assess the clinical diagnostic value of the newly developed strip, in comparison with that of a commercial real-time PCR assay.

## Results

### Conjugation optimization and characterization of antibody-gold/SWCNT

**Figure 1:**
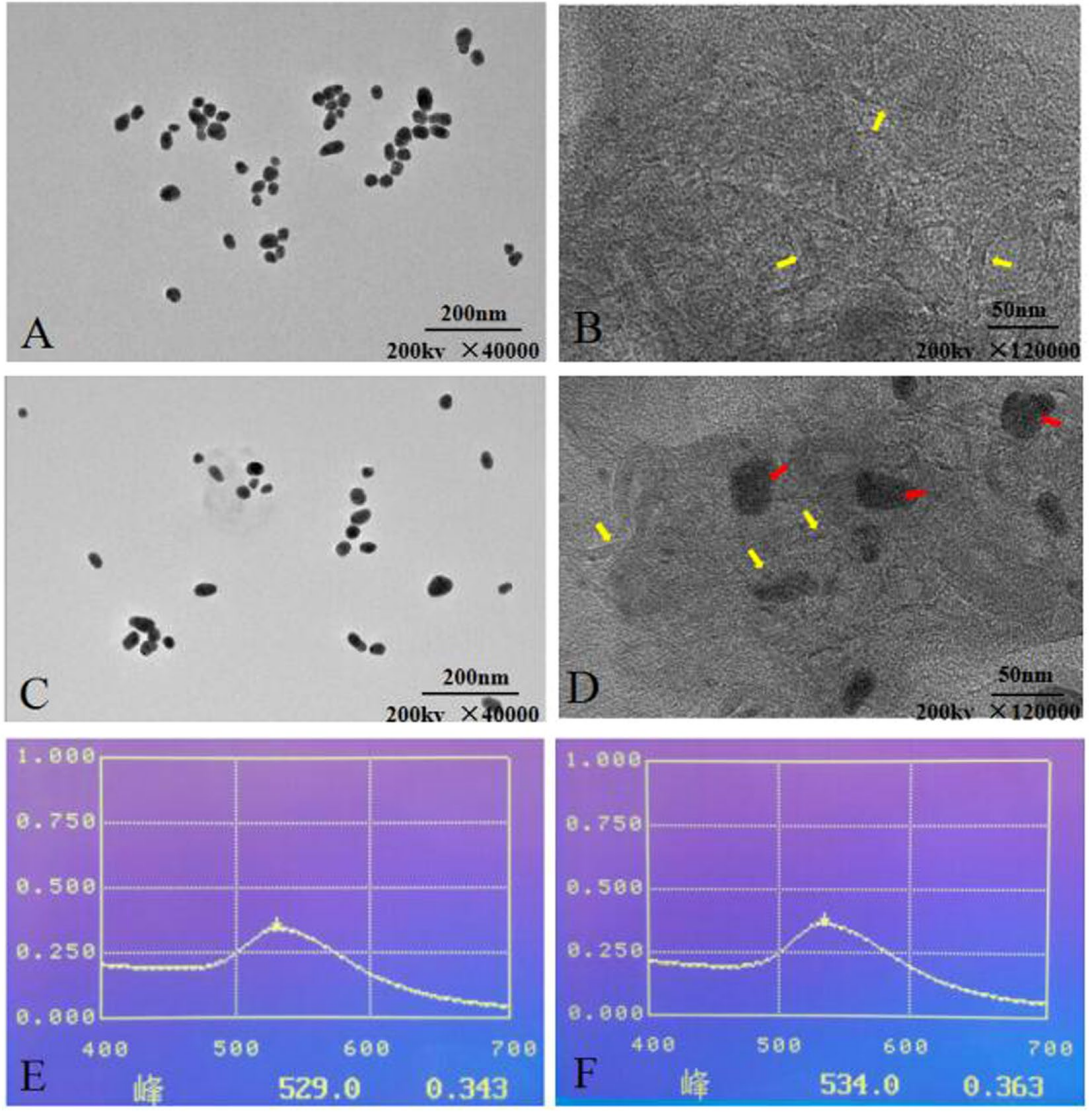
Characterization of antibody-gold/SWCNT. The TEM images of gold nanoparticle (**A**), SWCNT (**B**), antibody-gold conjugate (**C**) and antibody-gold/SWCNT (**D**). Yellow arrow:SWCNT, red arrow:gold nanoparticle. UV/Vis spectra of colloidal gold (**E**) and the antibody-gold/SWCNT conjugate (**F**).

The TEM images showed well-dispersed colloidal gold particles(Fig. 1A) and SWCNT (Fig. 1B). The average diameter of the colloidal gold particles was 28.95 ± 9.37 nm, which provided a good basis for preparation of CGIC. To stabilize colloidal gold particles, the optimum pH of antibody adsorption was determined to be 9.0. At this pH, 8 μg/mL capture antibody was confirmed to be the minimum amount for stabilizing colloidal gold solution. To ensure that enough antibody was used to conjugate with the gold particles and stabilize the colloidal gold, 10 μg/mL capture antibody was determined to be the optimum cencentration of monoantibody for the conjugation(Fig. 1C). The antibody-gold conjugates were adsorbed onto SWCNT and imaged using TEM (Fig. 1D). The TEM results were confirmed by UV/Vis spectra. According to the UV/Vis spectra of the colloidal gold and antibody-gold/SWCNT, there was a shift of peaks by antibody and SWCNT treatment. The peak at 529 nm of the colloidal gold curve was due to the surface resonance of colloidal gold particles. Added with the antibody and SWCNT, the surface resonance band shifted a little (Fig. 1E,F).

### *Mycoplasma pneumoniae* detection in the SWCNT/CGIC strip

**Figure 2:**
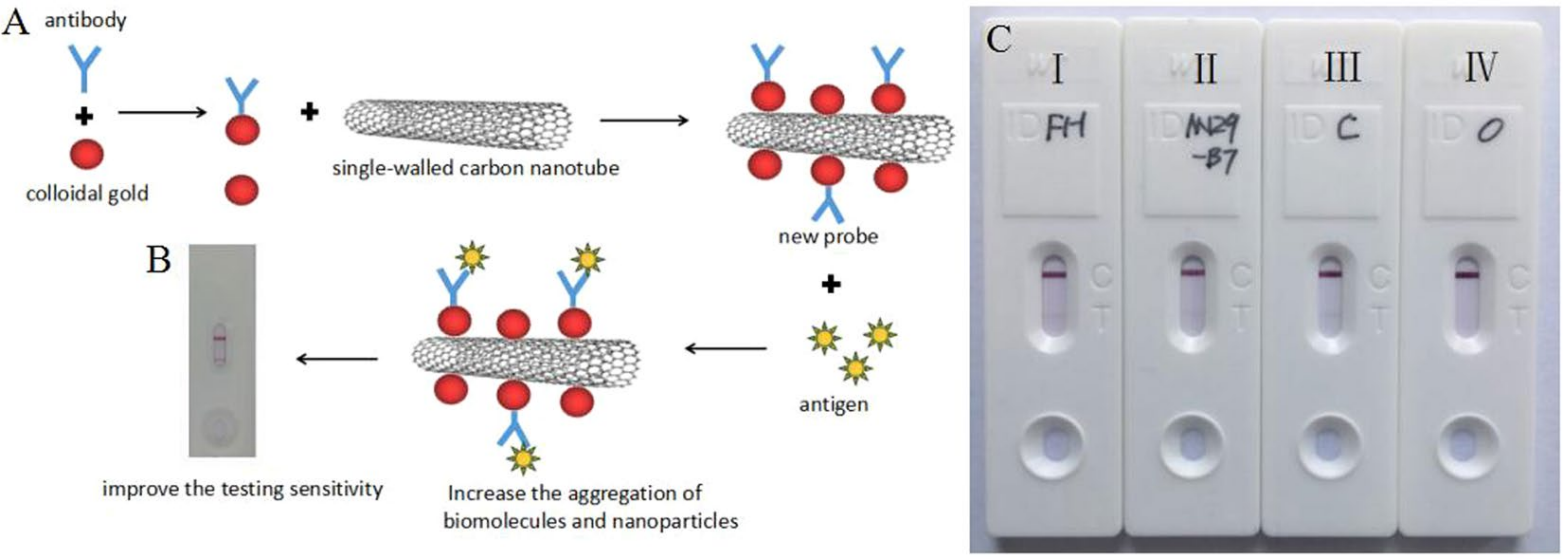
(**A**) Scheme of the SWCNT based immunochromatographic strips for MP detection. (**B**) SWCNT/CGIC strip setup is representative of a negative sample (left), and a positive sample(right). (**C**) FH (I,1 × 10^2^ copies/mL), M129 (II,1 × 10^2^ copies/mL) and isolates of MP (III, 1 × 10^2^ copies/mL) test results in the colloidal gold assays. IV:Negative control.

The principle of the single-walled carbon nanotube/colloidal gold-based immunochromatographic(SWCNT/CGIC) strip for *Mycoplasma pneumoniae* detection is illustrated in Fig. 2A. As shown in Fig. 2B, MP presence in a sample resulted in both the test and control lines being positive. A sample without MP displayed only a positive control line. To confirm the detection capacity of the colloidal gold assay, P1 genes of standard subtypes I(M129) and II(FH) *Mycoplasma pneumoniae* strains and one isolate of MP obtained from a patient were tested. The results showed that FH and M129 strains and isolates were positive in the SWCNT/CGIC assay (Fig. 2C).

### Comparison of SWCNT/CGIC strip and CGIC strip without SWCNT

**Figure 3:**
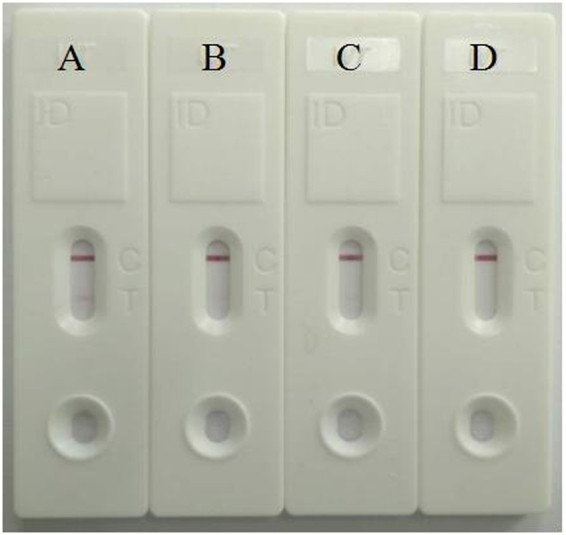
*Mycoplasma pneumoniae* detection. (**B**) Comparison the SWCNT/CGIC strip (**A**,**C**) with CGIC strip (**B**,**D** without SWCNT). (**A**) and (**B**) 1 × 10^3^ copies/mL, (**C**) and (**D**) 1 × 10^2^ copies/mL.

As shown in Fig. 3, the sample was recorded as positive if two clear red lines were observed. Different concentrations of MP samples (FH strain) were dropped onto the prepared strips. 1 × 10^3^ and 1 × 10^2^ copies/mL of MP samples(Fig. 3A,C) gave positive results using SWCNT/CGIC strip, 1 × 10^3^ copies/mL of MP sample(Fig. 3B) also gave positive result using CGIC strip without SWCNT, while 1 × 10^2^ copies/mL of MP sample(Fig. 3D) gave negative result using CGIC strip without SWCNT. These results showed that the SWCNT/CGIC strip had a higher sensitivity than the the conventional testing.

### Specificity, sensitivity and stability of the SWCNT/CGIC strip for *Mycoplasma pneumoniae*

To confirm the specificity of the strip,various conventional or atypical pneumonia pathogens were prepared by sample buffer, each pathogen at a concentration of 1 × 10^5^ copies/ml: *Mycoplasma pneumoniae*, *Haemophilus influenzae*, *Streptococcus pneumoniae, Staphylococcus aureus, Escherichia coli, Klebsiella Pneumoniae, Pseudomonas aeruginosa, Enterobacter cloacae, Staphylococcus epidermidis, Acinetobacter baumannii*, *Chlamydia pneumoniae*, adenovirus, respiratory syncytial virus, parainfluenza virus, influenza virus, human cytomegalovirus, human metapneumovirus and enteroviruses were assessed in 3 independent experiments. Our results showed that *Mycoplasma pneumoniae* was identified correctly by the assay with no cross-reactions found between MP and other pathogens (Data not shown).

**Figure 4:**
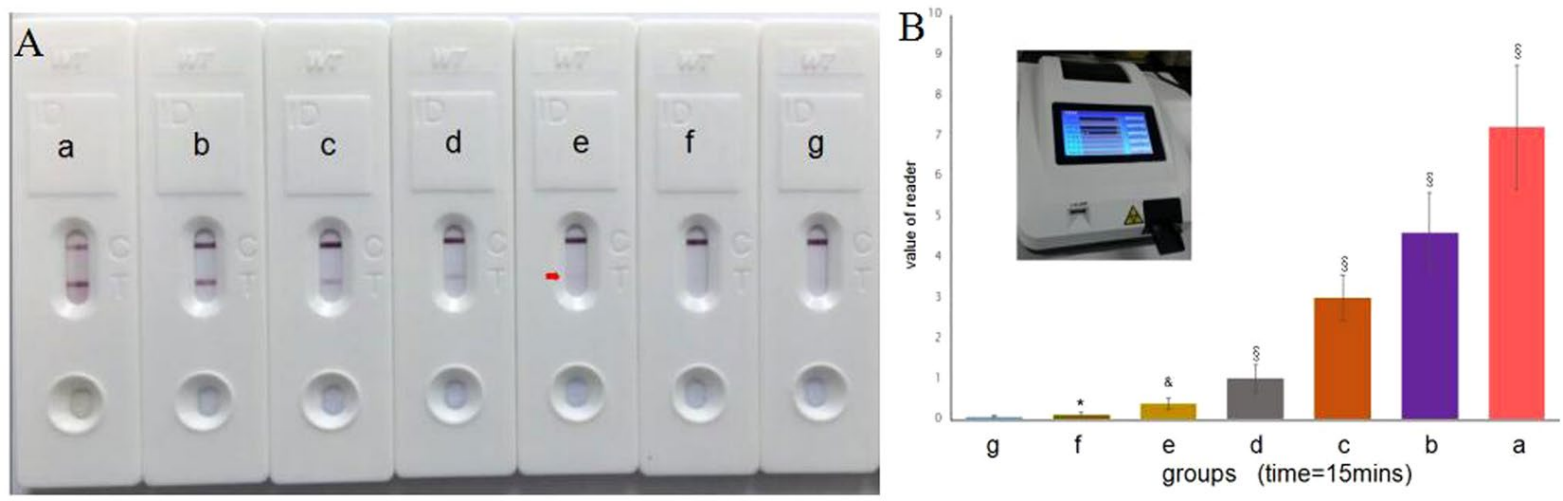
Sensitivity of the SWCNT/CGIC assay. (**A**) (a) 1 × 10^6^copies/ml; (b) 1 × 10^5^ copies/ml; (c) 1 × 10^4^ copies/ml; (d) 1 × 10^3^ copies/ml; (e) 1 × 10^2^ copies/ml; (f) 1 × 10^1^ copies/ml; (g) Negative control. (**B**) The intensity of red density on T lines was determined by using colloidal gold reading system. *P > 0.05; ^&^ *P* < 0.05; ^§^ *P* < 0.01 vs. Negative control (g).

To assess the sensitivity of the SWCNT/CGIC strip, standard *Mycoplasma pneumoniae* was quantified by real-time PCR and submitted to serial dilutions to obtain 10^1^ to 10^6^ copies/ml. Different concentrations of standard *Mycoplasma pneumoniae* were tested by the SWCNT/CGIC strip. As shown in Fig. 4A, MP with concentrations of 1 × 10^2^–1 × 10^6^copies/ml were positive in the assay (5 independent experiments). MP was not detected at 1 × 10^1^ copies/ml. The intensity of red density on T lines was determined by using colloidal gold reading system. This result of reader showed that the detection limit of the SWCNT/CGIC strip for MP was 1 × 10^2^ copies/ml (Fig. 4B). To evaluate the stability of immunochromatographic strips during storage, several of the immunochromatographic strips were stored for 3, 6, 12 and 18 months at room tempreature. The immunochromatographic strips left at room temperature for 12 months were not altered in terms of MP detection activity, whereas continual storage at room temperature for 18 months led to a reduction insensitivity.This demonstrated that the strips were stable for at least 12 months at room temperature(Data not shown).

### Effect of saliva, drugs on the SWCNT/CGIC strip

**Figure 5:**
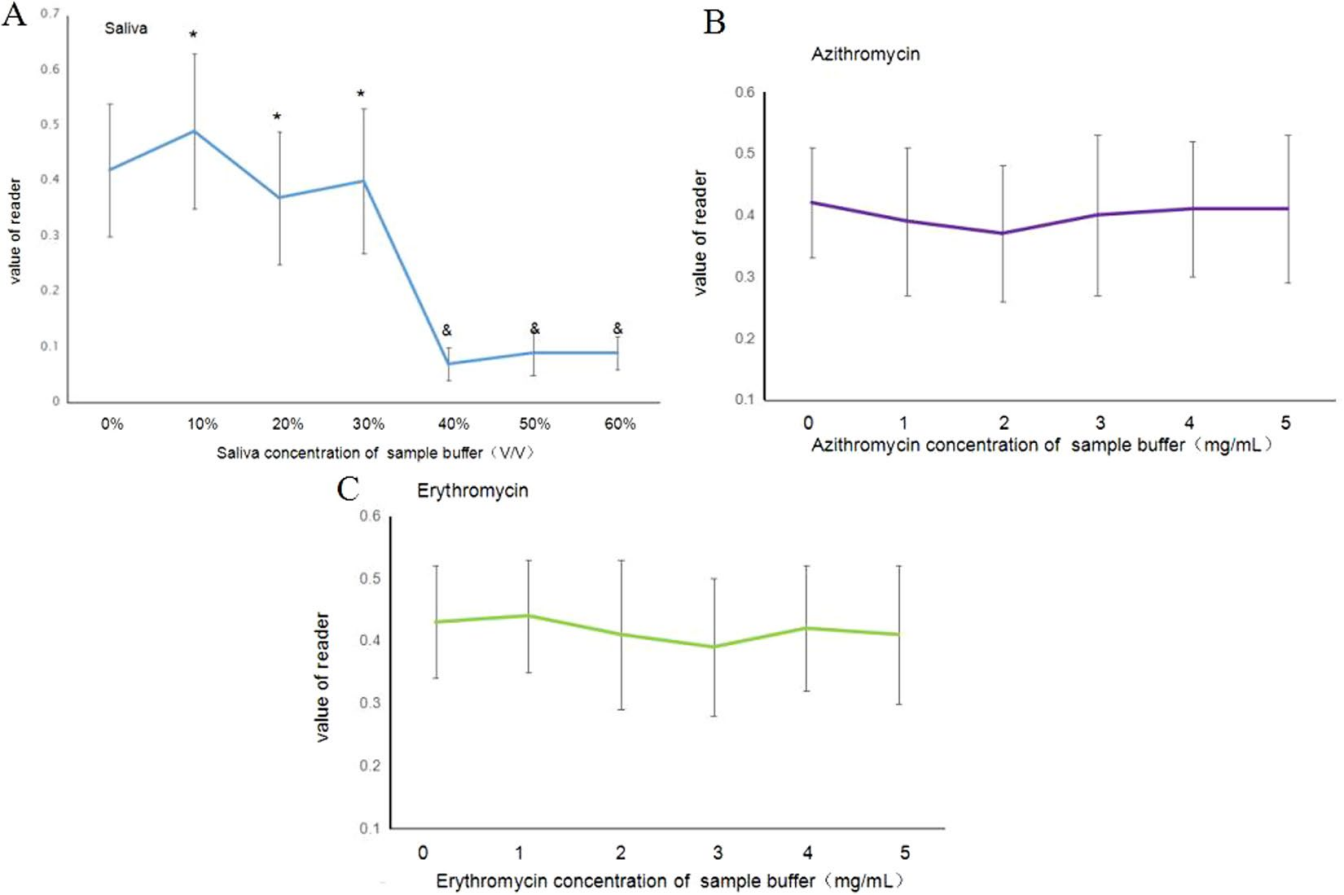
Effect of saliva, drugs on the SWCNT/CGIC strip. (**A**) Saliva (**B**) Azithromycin (**C**) Erythromycin (*P > 0.05; ^&^ *P* < 0.05 vs. Control group; MP = 1 × 10^2^ copies/mL,time = 15 mins).

To confirm the anti-interference ability of the strip, saliva and drugs of various concentrations were prepared by sample buffer. The results showed that at reasonable saliva(<30%, V:V) or drug(<5 mg/ml) concentrations, there was no effect on the detection of the SWCNT/CGIC strip for *Mycoplasma pneumoniae* (Fig. 5).

### Clinical specimen data

Among all the patients of MP group and Negative control group, 137 received MP SWCNT/CGIC assay. The results are shown in Table 1. PPV of SWCNT/CGIC assay was 100% and NPV was 59.7%. We concluded that the SWCNT/CGIC assay constructed by us had a high probability for confirming a diagnosis of *Mycoplasma pneumoniae* pneumonia and for guiding antibiotic choice for patients not yet treated.

**Table 1:** Sensitivity, specificity, and positive and negative predictive values of the SWCNT/CGIC assay for clinical specimens.

			SWCNT/CGIC assay	
			(+)	(−)
A combination of Real-time PCR and Clinical prediction rule	(+)	97	70	27
	(−)	40	0	40
	total	137	70	67
	Clinical sensitivity		70/97 = 72.2%	
	Clinical specificity		40/40 = 100%	
	PPV		70/70 = 100%	
	NPV		40/67 = 59.7%	

PPV = positive predictive value, NPV = negative predictive value.

## Discussion

*Mycoplasma pneumoniae* (MP) is a common pathogen of primary atypical pneumonia and other respiratory infectious diseases^14,15^. In addition, it is one of the most important agents of acute respiratory infections in children between the ages of 5 and 15 years. At present^15,16^, several methods are available for the diagnosis of infection, including culture, serological tests, and real time PCR techniques^17–19^. Real-time PCR and serological tests are currently used to diagnose MP clinically in China. But time-consuming and complicated protocols restrict their clinical application. Our SWCNT/CGIC assay offers a sensitive and specific tool for rapid and simultaneous detection of MP.

Colloidal gold-based immunochromatographic assay has been confirmed to be a rapid and sensitive method for serological surveillance and detection of bacterial pathogens in the field^20^. In this study, we applied SWCNT/CGIC assay to detect MP by targeting the specific P1 antigen. The membrane protein P1 is thought to have a major receptor-binding role in MP^21^. Clinical MP strains can be divided into two types (I and II) according to their P1 gene variants^22,23^. The SWCNT/CGIC assay has high capacity to detect both types of MP. MP detection could be completed in 10 minutes by using our method, while real-time PCR requires 2 to 4 hours, and culture usually takes 21 days or more^19,24^.

This work clearly reveals that single-walled carbon nanotubes can be employed for the successful design of platforms for immobilization of colloidal gold-antibodies suitable to be used as rapid analytical tools with potential application in the diagnosis of MP. Single-walled carbon nanotubes have a large surface area to volume ratio which makes them immobilize more colloidal gold-antibody conjugations to increase the sensitivity of the strip. Moreover, this method presents high specificity in detecting MP, no cross-reactions with other clinical pathogens.

The detection limit of SWCNT/CGIC assay (1 × 10^2^ copies/mL) for MP was comparable to that of the Asahi company rapid antigen kit, which demonstrated sensitivities of 8.3 × 10^4^ copies/mL^25^. In clinical test, we used commercial real-time PCR assay as a control method, targeting the P1 gene of MP. We applied the newly developed SWCNT/CGIC assay to the 137 specimens from children with pneumonia. 70(51.1%) specimens were positive for MP. When compared with real-time PCR, the specificity and sensitivity of the SWCNT/CGIC assay were 100% and 72.2%, respectively. The symptoms in all MP positive children were improved after treatment with azithromycin.

Reliable, rapid, and easy-toperform tests for the diagnosis of MP infections are helpful in guiding azithromycin usage and preventing further selection of macrolide-resistant MP.

In conclusion, we have successfully developed a new immunochromatographic strip for the rapid detection of the MP antigen using colloidal gold coupled with monoclonal antibody/single-walled carbon nanotubes, with high sensitivity and specificity. The new method is, therefore, a ideal and rapid determination method for detection of MP. The colloidal gold probe, colloidal gold coupled with monoclonal antibody/single-walled carbon nanotubes, we developed in this study can easily be extended for detecting other bacterial cells in real samples with high accuracy and sensitivity.

## Methods

### Materials

Chloroauric acid (HAuCl_4_·3H_2_O) and sodium citrate were purchased from the company of Sigma (USA). Bovine serum albumin (BSA) and goat anti-mouse IgG were purchased from DAKO (DK). High-flow nitrocellulose membrane, glass fiber, and absorption pad were purchased from Millipore (Bedford, MA). The mouse momoclonal antibodies against MP (named P-Ab1501 and P-Ab1502) were prepared by Benxi TestJet Biotechnologies. Bovine serum albumin (BSA) and goat anti-mouse IgG were purchased from Sigma (USA). Single-walled nanotubes were purchased from Beijing DK Daoking Technology Co.Standard *M. pneumoniae* FH (ATCC 15531) and M129 (ATCC 29342) strains were purchased from ATCC. *Haemophilus influenzae*, *Streptococcus pneumoniae, Staphylococcus aureus, Escherichia coli, Klebsiella Pneumoniae, Pseudomonas aeruginosa, Enterobacter cloacae, Staphylococcus epidermidis, Acinetobacter baumannii*, *Chlamydia pneumoniae*, adenovirus, respiratory syncytial virus, parainfluenza virus, influenza virus, human cytomegalovirus, human metapneumovirus and enteroviruses were provided by Benxi TestJet Biotechnologies(China). All other chemicals used in the present study were analytically pure.

### Clinical specimens

From Dec 1,2016 to Jan 31,2017, 137 children were enrolled in this study. The inclusion criteria were: (1) 3 <age< 14 years (56 females and 81 males); (2) patient visiting the pediatric respiratory depatment of The First Hospital of Jilin University; (3) primary diagnosis as pneumonia according to known guidelines(China). During 3 months, 137 specimens were collected. Each specimen was mixed with 1.0 ml sample buffer and stored at −70 °C; 0.5 ml of the mixture was used for real-time PCR and 0.5 ml in the SWCNT/CGIC assay.

The study was performed in accordance with the Declaration of Helsinki and approved by the Medical Ethics Committee of The First Hospital of Jilin University. All patients provided informed consent.

### Synthesis and characterization of colloidal gold

In brief, the pH of the colloidal gold solution was adjusted to pH around 9.0 by adding 0.2 mol/L K2CO3 before the mouse monoclonal antibodies (named P-Ab1502,against *M. Pneumoniae*, which target the *M*. *pneumoniae* P1 antigen,were prepared by Benxi TestJet Biotechnology Co.) were added. Ten mL of colloidal gold was mixed with 200 μL of 0.5 mg/mL antibody, with shaking at intervals for 30 min.Then, another 1000 μL of 5% BSA was added, with shaking at intervals for 30 min. The dispersion of SWCNT (10 mg, DKnano, China) in pure water (100 mL) by ultrasonication for 5 min in presence of Tween20 (0.1 mL). The solution of Colloidal gold/IgGs was mixed with 50 μL SWCNT, with shaking at intervals for 30 min. The mixture was centrifuged at 6275 × g for 30 min at 4 °C. The pellet (SWCNT-colloidal gold-labeled antibody conjugation) was re-suspended with 5000 μL of the suspension buffer (0.01 mol/L pH 8.5 Tris-HCl, 25% sucrose, 2.5% BSA, 1.5%Tween-20, 0.25%PEG20000, 0.20%sodium caseinate). The mixture was centrifuged at 6275 × g for another 30 min at 4 °C, and the pellet was re-suspended in 240 μL of suspension buffer. SWCNT-colloidal gold-labeled antibody conjugation was jetted onto glass fiber and dried at 37 °C for 4 h.

The antibody against MP (P-Ab1501 antibody) was diluted to 1.5 mg/mL with 0.01 mol/L phosphate-buffered (PB; pH7.4). The diluted antibody (Test line, T line) and goat anti-mouse antibody (Control line, C line) were transferred onto a nitrocellulose membrane, which was then dried for 2 h at 37 °C.

The SWCNT/CGIC strip was composed of a sample pad, a conjugate pad, an immobilized nitrocellulose membrane, and an absorbent pad.

Colloidal gold particles were examined by a transmission electron microscope (TEM) performed on a JEM-2100 (JEOL, Japan) and operated at an acceleration voltage of 200 kV.

### Real-time PCR for MP detection

For real-time PCR, 0.5 ml of the mixture was centrifuged for 5 min at 12000 rpm/min. The cell pellets were resuspended in 50 μL lysis buffer (Da An Gene Co., Ltd., China); 4 μL lysate served as template in real-time PCR amplification based on the TaqMan probe PCR kit (Da An Gene Co., Ltd., China) according to the manufacturer’s instructions. Real-time PCR was carried out on an ABI 7500 instrument for 3 min at 95 °C, followed by 40 two-step cycles (15 s at 95 °Cand 45 s at 55 °C).

### MP serology analysis

Diagnostic Kit for Measurement of Antibodies to Mycoplasma pneumoniae (Passive Particle Agglutination, PPA)(SERODIA-MYCO II, Japan) was used to dectect the IgM antibodies. The plates were shaken for 30 s and then covered and left undisturbed on a level surface at room temperature for 3 hours. Results were considered positive at 1/80 titer or more.

### SWCNT/CGIC assay for MP detection

To perform the SWCNT/CGIC assay, 80 μL (about 2 drops) of the mixture was added into a sample well for 10–15 minutes. Samples with positive control and test lines were determined as MP positive; no Control line on the plate indicated an invalid test.

### Statistical analysis

The results were expressed as mean values with their corresponding standard errors. One-way analysis of variance (ANOVA) with least significant difference test (LSD) was used to compare experimental groups. A calculated *P* < 0.05 was considered statistically significant. All analyses were performed by SPSS for Windows Software Version 17.0.
